# Risk of mental health and nutritional problems for left-behind children of international labor migrants

**DOI:** 10.1186/s12888-015-0412-2

**Published:** 2015-03-06

**Authors:** Kolitha Wickramage, Chesmal Siriwardhana, Puwalani Vidanapathirana, Sulochana Weerawarna, Buddhini Jayasekara, Gayani Pannala, Anushka Adikari, Kaushalya Jayaweera, Sharika Peiris, Sisira Siribaddana, Athula Sumathipala

**Affiliations:** 1 Health Unit, International Organization for Migration, No. 62, Green Path, Colombo 3, Sri Lanka; 2 Institute for Research & Development, Colombo, Sri Lanka; 3 Faculty of Medical Science, Anglia Ruskin University, Chelmsford, UK; 4 Institute of Psychiatry, King’s College London, London, UK; 5 Department of Medicine, Faculty of Medicine & Allied Sciences, Rajarata University of Sri Lanka, Mihintale, Sri Lanka; 6 Ministry of Health, Colombo, Sri Lanka; 7 Research Institute for Primary Care and Health Services, Faculty of Health, Keele University, Keele, UK; 8 Department of Cardiothoracic Vascular Surgery, National University Hospital, Singapore, Singapore

**Keywords:** Migrant workers, Child mental health, Left-behind families, Sri Lanka

## Abstract

**Background:**

One-in-ten Sri Lankans are employed abroad as International Labor Migrants (ILM), mainly as domestic maids or low-skilled laborers. Little is known about the impact their migration has on the health status of the children they ‘leave behind’. This national study explored associations between the health status of ‘left-behind’ children of ILM’s with those from comparative non-migrant families.

**Methods:**

A cross-sectional study design with multi-stage random sampling was used to survey a total of 820 children matched for both age and sex. Socio-demographic and health status data were derived using standardized pre-validated instruments. Univariate and multivariate analyses were used to estimate the differences in mental health outcomes between children of migrant vs. non-migrant families.

**Results:**

Two in every five left-behind children were shown to have mental disorders [95%CI: 37.4-49.2, p < 0.05], suggesting that socio-emotional maladjustment and behavioural problems may occur in absence of a parent in left-behind children. Male left-behind children were more vulnerable to psychopathology. In the adjusted analyses, significant associations between child psychopathological outcomes, child gender and parent’s mental health status were observed. Over a quarter (30%) of the left-behind children aged 6–59 months were ‘underweight or severely underweight’ compared to 17.7% of non-migrant children.

**Conclusions:**

Findings provide evidence on health consequences for children of migrant worker families in a country experiencing heavy out-migration of labour, where remittances from ILM’s remain as the single highest contributor to the economy. These findings may be relevant for other labour ‘sending countries’ in Asia relying on contractual labor migration for economic gain. Further studies are needed to assess longitudinal health impacts on the children left-behind.

## Background

International labor migration has become a crucial engine for economic development for many countries worldwide [1,2]. The growing economic aspirations of International Labor Migrants (ILM’s) are driven by labor market demands of rapidly developing regions of the world. ‘Source’ countries for migrants from Asia, particularly female domestic maids include Sri Lanka, Philippines, Pakistan, Indonesia, India and Bangladesh [3]. ‘Destination’ countries consist of mainly the Gulf States, followed by emerging economies in Asia such as Singapore.

ILMs from Sri Lanka has grown ten-fold during the past decade, with 90% employed in the Gulf, and 730 registered workers departing Sri Lanka each day [4]. In what was once a highly feminized labor force, today 49% percent of ILMs are women, and of these 86% are employed as domestic housemaids [4]. Remittances by migrant workers remained the single highest contributor to the Sri Lankan economy in 2012, with earnings expected to increase to 7bn USD by 2016 [5]. Despite these clear monetary benefits for the State, studies examining household savings and socio-economic status of returning Sri Lankan ILMs show mixed individual economic gains [6,7]. Due to this, most workers choose continuous cycles of re-migration (circular migration) to increase their savings. The ‘balance sheet’ of labor migration typically involves a trade-off between economic well-being and family proximity [8,9]. Through an economic lens, remittances may directly benefit a majority of poorer migrant households by increasing income. However, the reliance on remittance alone as a measure of poverty alleviation remains unclear. Its utility for human capital formation in migrant households through greater spending on health care, food and education also requires empirical exploration [7,10].

United Nations (UN) agencies have articulated the need for migration-related determinants of health to be explicitly included in the post-2015 Millennium Development Agenda through the UN general assembly’s High Level Dialogue on International Migration and Development, and through the Global Migration Group [11]. The International Organization for Migration (IOM) and the World Health Organization (WHO) have led global efforts to stimulate member states to adopt migrant sensitive health systems and enable policies and practices to ensure realization of the right to health for migrant and mobile populations [12]. The UN Committee on the rights of the child have advocated for protection of child rights in the context of international migration, and the United Nations Children Fund (UNICEF) has also articulated the scarcity of research on consequences of migration on child health and wellbeing worldwide [13].

### Effects on child psychological well-being

The adverse effects on the psychological well-being and development outcomes of children due to separation from a parent for extended periods has been characterized by a number of researchers [14-17]. However, such research examining transnational parental effects has focused on immigrant groups within industrialized countries rather than temporary labor migrant populations [18,19]. Only a few studies have examined the psychological impact on children of temporary labor migrant families from developing nations. A study by Graham and Jordan [20] measured psychological well-being among children of labor migrants under the age of 12. They showed that children of households in Indonesia and Thailand where mothers were labor migrants had poorer psychological well-being indices than children from non-migrant households [20]. A study of school children in Philippines by Battistella and Conaco [21] found little or no evidence that children of migrant families had greater psychological problems than children of non-migrants.

Three studies from Sri Lanka examined health status of left-behind children utilizing standardized psychometric measures [22-24]. Results from all three indicated that absence of the mother was significantly associated with adverse behavioural problems in left-behind children. However, the following limitations were common in all the studies. First, they focused exclusively on male-headed households (where the mother was the overseas worker) and did not include female-headed households. Considering that 52% of migrant workers are male [4], this left a significant gap in assessing health impact of children in such families. All studies used purposive samples obtained entirely from selected schools in an urbanized setting from a single district, and children of only one ethnic group (Sinhalese) were included. In order for policy makers and planners to make informed decisions on the impact of migration on childrens’ well-being, more representative studies are needed from areas that supply large number of ILM’s.

### Effects on child nutrition

A number of studies have investigated the relationship between international labor migration and child malnutrition [25-27]. Remittances flowing from ILM’s may affect child nutrition through two broad pathways. First, increased household income from the migrant parent sending back remittances may be used to enhance purchasing power for food and other goods. Secondly, by changing time and task allocations within the household, as the loss of a parent may reduce the time available to prepare food and/or to care for the child’s nutritional needs. A review of literature identified only a few studies that examined nutritional outcomes in children left behind due to ILM. A study by Cameron and Lin [28] highlighted that the absence of a parent in migrant households had a negative effect on short-term child nutrition in Thailand. However, authors suggested increasing levels of household remittances may help lessen the negative effect on child nutrition. A nationally-representative longitudinal study by Nobels [29] in Mexico examined the effect of parental migration on child health by comparing children within households who are exposed to migration at critical periods of child development. Results suggested that parental migration negatively effects child height-for-age, a long-term measure of child nutritional status and illness. The same pattern did not emerge from comparisons of children in non-migrant households. Frank and Hummer [26] who also studied Mexican migrant and non-migrant households found that membership in a migrant household reduced the risk of low birth weight, largely through the receipt of remittances. Though few in number, these studies highlight the complex relationship between temporary labor migration and child nutrition.

With high rates of malnutrition in developing nations that are also major source countries for ILMs, and with the growing evidence base linking child nutritional deficiencies with mental health problems [30,31] exploring child nutrition remains an important policy area.

### Research objective

Despite the political discourse on migration moving up the global development agenda [1,12], the public health implications for migrants and their families have received little attention. Analysts have also argued that Global Migration policy strategies have failed to recognize and adopt a family perspective [32,33]. A PLoS medicine series on *Migration & Health* in 2011 prompted public health attention and called for an evidence-based research agenda on health of migrants [34]. As described, there have been relatively few studies from countries, which ‘supply’ labor that considered the effects of international labor migration on children left-behind. This paper addresses the question of whether migrant children face an increased risk for adverse mental health and nutritional outcomes in Sri Lanka.

## Methods

### Study design and participants

A cross-sectional survey was conducted in six districts of Sri Lanka with the highest number of outbound ILMs. The study population included the families of migrant workers (employed abroad for at least six months), residing in one of the selected districts. The inclusion criteria for the study group were households where one or both parents were ILMs, who had their own or adopted child/children under 18 years of age living at the same residential address for a period of (at least) six months prior to the time of data collection. Children from families without a history of migration abroad were considered as the non-migrant ‘comparative group’. The inclusion and exclusion criteria was adopted to ensure an accurate comparison of the effect of migration on left-behind families of ILMs is presented below. Our study included analysis of both adult and child members from these families, however, this paper only describes the child sample. We also undertook a comprehensive qualitative research study to explore the perceptions of left-behind migrant families, which has been published separately [35].

### Definitions of participant categories and their inclusion and exclusion criteria

- *Migrant Family*: Inclusion criteria: a family where either one or both spouses have departed for employment abroad as a labour migrant for period of at least six months, have their own or adopted child/children under 18 years of age, and the left-behind family been living at the same residence for a period of at least six months at the time of data collection. Exclusion Criteria: families in which the migrant worker was continuously absent in the preceding six months prior to leaving the country on assignment.
- *Migrant Spouse*: the spouse of the overseas-based migrant worker living in the migrant family household for at least six months.
- *Child ‘left-behind’ (or “left-behind child’)*: a child under 18 years (at the time of data collection) who is living in the migrant family household for a period of at least last six months, and who’s parent/parents are international labour migrant workers currently working abroad for a period of at least 6 months.
- *Caregiver*: a person living in the migrant family household who is not the biological mother/father, but who is responsible for taking on the burden of care for the left-behind child on a daily basis, for a period of at least six months. Care consists of activities such as; arranging daily schedules, preparing or ensuring access to meals, assisting the child’s educational and social needs (including play), washing clothes, looking after the child when he/she is sick, guardianship and representation to health and/or education authorities.
- *Comparative (Non-Migrant) Family:* Inclusion criteria: A family where both parents are present, which neither spouse has a history of labour migration (both internal and outbound), have their own or adopted child/children under 18 years of age in the family unit. Exclusion criteria: one or both parents being absent from the same house for more than 60 days (average more than 2 days per week) continuously or alternatively for the preceding six months

### Sampling

A multi-stage random sampling method was used in the selection of ILM households. Grama Niladhari Divisions (GND) or ‘village unit’ is the smallest administrative population unit in Sri Lanka. Divisional Secretariat Divisions (DSD) administrates a cluster of GNDs, and is responsible for coordinating social services in these villages [36]. A total of 41 DSDs were included in six districts with the highest number of ILMs (Colombo, Gampaha, Kandy, Kalutara, Kurunegala and Puttalam). All GND in each 41 DSDs were listed and one GND was randomly selected from each DSD using a random number generation tool totaling 41 GNDs. A registry of all migrant households in each selected GND was created according to the information obtained from the Grama Niladhari (village administrator), Public Health Midwife (PHM), and *Samurdhi Niyamaka* (government appointed village welfare worker). Subsequently, ten migrant family households were selected randomly from each GND. Each randomly selected household was checked for inclusion and exclusion criteria, and to compensate for ineligible households, another household was selected randomly from the remaining list in the GND. The sample size was estimated using standard sampling power calculations [37].

A total of 410 migrant worker families and 410 families with no migration experience were included in study. Children between 12 to 17 years of age from migrant households were individually matched with children from the same school and class to find a comparative child from a non-migrant household within each GND. Children were then matched according to gender and age, and a list of children from families with no migration history were obtained from the attendance register available in the classroom with the responsible teacher. Similarly, pre-school children (under 5 years of age) were also matched according to gender and age, from the list obtained from the PHM registry.

Of the six districts, Colombo and Kandy have the vast majority of population living in densely populated urban centres with some peri-urban zones. Kurunegala and Gampaha districts have a population within a mix of urban, peri-urban and rural population catchments. Population in Puttalam district is concentrated in predominantly rural settings, with small urban centres. Rural populations are characterized by their dependence on agriculture for their livelihood, with an estimated 90% of the nation’s poor living in rural settings.

### Standardized health instruments and outcome measures

The study instruments were extensively validated using nominal group techniques and were previously used by authors in a large-scale national study on adult and child mental health in Sri Lanka [38].

The *Questionnaire for socio-demographic data* aimed at capturing basic social, economic, environmental and demographic indicators. Variables included gender, ethnicity, family size, employment type, educational status, home ownership status, household setting/conditions, household goods, income and expenditure. Measures such as migration history, frequency of ILM return from country of labour migration, household indebtedness, and frequency of remittance sent home were also captured. The questionnaire was administered to both the spouse/caregiver of the migrant family and to one adult member (parent of selected child) of the comparative non-migrant family.

The *Strengths and Difficulties Questionnaire (SDQ)* is a reliable measure of the adjustment and psychopathology of children and adolescents. It indicates emotional symptoms, conduct problems, hyperactivity/inattention, peer relationship problems and pro-social behaviour. A computerised predictive algorithm generates “unlikely”, “possible” or “probable” ratings for four broad categories of disorders, namely conduct disorder, emotional disorder, hyperactivity disorder, and any psychiatric disorder. The algorithm has been deemed to be sufficiently accurate and robust to be of practical value, and the level of chance-corrected agreement between SDQ prediction and independent clinical diagnosis considered substantial and highly significant (Kendall”s tau-b between 0.49 and 0.73; p < 0.001) [39]. The composite score from both ‘abnormal’ and ‘borderline’ scores were calculated to assess the risk potential in left-behind children to develop psychopathology. A ‘borderline’ or probable SDQ prediction for any given disorder correctly identified 81-91% of the children who definitely had that clinical diagnosis [39].

The three versions of the SDQ, for children, parents and teachers were used in the study. The instrument was adapted from original screening questionnaires [39], translated to both Sinhalese and Tamil languages and identical to one successfully used in a national child mental health survey in Sri Lanka [38]. The SDQ versions for teachers, parents, and for children between 6 to 17 years of age were administered to both migrant and comparative families. For each selected child or adolescent between the ages of 6 to 11 years, the child’s parent/care-giver and their schoolteacher completed the SDQ, assisted by a trained field researcher. Children between the ages of 12 to 17 years also completed the SDQ and the composite score triangulated with results obtained by their parent and schoolteacher.

The *Check list for growth development and immunization (CHDR)* was used to collect data from children under 5 years of age that were matched by age and gender from both migrant and comparative families. We focused on capturing nutritional status of young children aged 0 to 5 years, as these formative years are crucial for child growth and development [40]. Anthropometric data on child’s growth, development milestones and immunization history were captured from individual Child Health Development Records (CHDR) issued by the Ministry of Health. PHM at the village level regularly recorded these measures at child health clinics, and records were also held by the parent/care-giver of the child. The CHDR registers the growth for children from birth to 5 years of age by body mass relative to age (Weight-for-age). Weight-for-age (WFA) is commonly used for monitoring growth to assess changes in the magnitude of malnutrition over time. A child’s ‘underweight’ status reflects both chronic and acute malnutrition (underweight is defined by a WFA Z-score between < −2 and ≥ −3 SD from mean). Child nutritional status (using Z-score measures) and immunization history were recorded from CHDR records maintained by PHM from birth.

### Ethics, data collection & analysis

The Ethics Review Committee of the Faculty of Medicine, University of Colombo, granted ethical approval. Data collection was conducted using a team of 22 trained field research assistants under the guidance of a psychiatrist, physician and two public health specialists. Permission to collect data was obtained from the regional education authorities, principals and teachers of each school. Participant information leaflets were sent to the parents through the selected school children. Later, the study was fully explained and written consent obtained from the parent or guardian. The study was also explained to all children and their assent was obtained.

Data collection was supervised and managed by two dedicated project coordinators and a statistician. Double data entry and data analysis was conducted using SPSS (Statistical Package for Social Sciences) version 17 and STATA. Statistical analysis included descriptive analysis to determine demographic information and frequency of the exposure and outcome variables. Chi-square tests were performed to ascertain differences between migrant and comparative children. We used standard multivariable linear regression models for continuous outcomes and multivariable logistic regression models for dichotomous outcomes. Univariable and multivariable analyses were used to investigate psychological outcomes of children of migrant vs. non-migrant families. Multivariable models were adjusted for child age and gender.

## Results

### Socio-demographic characteristics

A total 410 children from migrant families were matched with 410 children from comparative households (228 children were less than 5 years of age, and 592 were aged 12 to 17 years). A response rate 94% (n = 770) was achieved from the total of 820 families that were recruited for the study (385 children from migrant families were matched with 385 children from comparative households) (Table 1). A similar gender disaggregation profile was observed in children of both migrant and non-migrant families (49.4% for males and 46.8% for females). Vaccination status according to the Ministry of Health guidelines of the expanded program in immunization schedule (EPI) showed a high level of coverage, with the migrant children having a 95.4% completion rate.

**Table 1:** **Demographic characteristics of children from migrant and comparative non-migrant households**

	Left-behind children (%)	Comparative children (%)	Group difference (chi ^ 2 ^ , df, p-value)
***Age***			0.12 (3), p = 0.950
1-4 yrs	77 (20.0)	83 (21.6)	
5-8 yrs	104 (27.0)	99 (25.7)	
9-12 yrs	91 (23.6)	91 (23.6)	
13-17 yrs	113 (29.4)	112 (29.1)	
***Gender***			0.52 (1), p = 0.471
Male	195 (50.6)	205 (53.2)	
Female	190 (49.4)	180 (46.8)	
***Vaccination status*** ^**a**^			0.65 (1), p = 0.422
Completed	103 (95.4)	103 (92.8)	
Not completed	5 (4.6)	8 (7.2)	
***Children with special needs*** ^**b**^			1.41 (1), p = 0.236
Need of special care	5 (4.6)	2 (1.8)	

^a^Vaccination status of children under 5 years according to National Expanded Program in Immunization (EPI) schedule.

^b^Children with special needs are defined as those having a chronic physical, developmental, behavioral, or emotional condition and require health and rehabilitative services of a type or amount beyond that required by children generally.

### Nutritional status of children under 5 years of age

The measure of ‘underweight’ contained in the CHDR reflects both chronic and acute malnutrition, and is measured by weight relative to age. The proportion of children that were in normal weight range (z-score of < + 2 to – 1 SD) in the migrant households was 38.2%, while the frequency in comparative households was higher at 46.9% (Table 2). Over a quarter (30%) of the left-behind children were underweight or severely underweight, compared to 17.7% of non-migrant children. However these effects were not statistically significant (p = 0.061).

**Table 2:** **Nutritional and psychological status of children from migrant and comparative non-migrant households**

	Left-behind Children (%)	Comparative Children (%)	Group difference (chi ^ 2 ^ , df, p-value)
***Nutritional status of children aged 6–59 months*** ^**a**^			2.28 (4), p = 0.061
Overweight	4(3.6)	3(2.7)	
Normal weight	42(38.2)	53(46.9)	
Risk of Underweight	31(28.2)	37(32.7)	
Underweight	27(24.5)	20(17.7)	
Severely underweight	6(5.5)	0	
***Child psychopathology scores (SDQ Domains)*** ^**b**^			
Emotional problems	30 (10.9)	9(3.4)	6.60 (1), p = 0.010
Conduct problems	108(39.3)	84(31.3)	3.75 (1), p = 0.053
Hyperactivity disorder	22(8.0)	8(3.0)	11.82 (1), p = 0.001
Any psychiatric diagnosis	119(43.3)	90(33.6)	5.42 (1), p = 0.020

^a^Underweight reflects both chronic malnutrition and acute malnutrition. It is measured by weight relative to age (WFA). Normal weight is defined as a WFA z-score of + 2 to – 1 SD. Underweight is defined for a z-score of < −2 and ≥ −3 SD. Severely underweight a z-score < −3 SD. Overweight represents excessive fat accumulation that presents a risk to health, and is measured by calculating the child’s Body Mass Index against their age - labelled ‘weight for height’ (WFH). The range for ‘overweight’ is a z-score > +2 and ≤ +3 SD.

^b^The SDQ domain ‘any psychiatric diagnosis’ aggregates emotional, conduct and behavioural scores to provide a potential measure a person has to develop or have a psychiatric disorder.

### Mental health burden of children

The dimensions of child psychopathology (emotional problems, hyperactivity disorder, conduct problems and having any psychiatric diagnosis) of children 6 to 17 years of age was measured through the Strengths and Difficulties (SDQ) scale, and predicted children of migrant families would experience greater emotional problems following parent–child separation than non-migrants. The burden of emotional problems [10.9%, 95%CI: 7.2 -14.6, p < 0.05] and hyperactivity disorders [8%, 95%CI: 4.7-11.2, p < 0.005] is highest in children from migrant families than from children in comparative families (Table 2). Two in every five left-behind children (43.3%) had clinically relevant child psychiatric disorders [95%CI: 37.4-49.2, p < 0.05]. Whilst child conduct problems were higher in left-behind children (39.4%) than those in non-migrant households (31.3%), these were not statistically significant (p = 0.053).

The mental health status of the parent/caregiver of a child was strongly associated with child psychopathology. This effect was observed in both migrant [Adjusted OR 1.63 (95%CI: 0.89-2.96)] and non-migrant families [Adjusted OR 2.44 (95%CI: 1.03-5.80), p < 0.005].

### Migration and health related factors of parents and caregivers

The ethnic profile of the study sample closely matched national population ratios from the 2001 national population census, with 74.5% of migrant families and 78.2% of non-migrant groups being of Sinhalese ethnicity [41]. The mean age of parents of left-behind children was 37.9 years, with the majority of families living within rural settings (69%). There were twice as many fathers taking the role as the primary carer of left-behind children in the migrant family group (28.8%) than of the comparative non-migrant group (13.2%)(p < 0.001). The results show the proportion widowed/divorced parent to be greater (3.7 fold) in the migrant family group than in comparative families (p < 0.001).

Typology of employment of the migrant worker was assessed according to the Sri Lanka Bureau of Foreign Employment (SLBFE) classification of occupations [4]. The majority (66%) belonged to the low-skilled occupation classification of ‘manual laborers’ and ‘domestic housemaids’ (Table 3). More than half (55.6%) of migrant workers were reported by their spouses as having not returned to Sri Lanka since going abroad for work. Only 42.9% of the spouses of migrant workers reported receiving some form of monthly monetary remittances.

**Table 3:** **Socio-demographic, migration and health related characteristics of parents in migrant and non-migrant households**

	Migrant spouse (%)	Comparative spouse (%)	Group difference (chi ^ 2 ^ , df, p-value)
***Gender***			18.64 (2), p = 0.001
Male	111 (28.8)	51 (13.2)	
Female	274 (71.2)	334 (86.8)	
***Age***			29.14 (1), p = 0.001
Spouse age (mean)	37.9 (0.49)	37.0 (0.39)	
18-30	54 (14.1)	77 (18.3)	
31-60	293 (76.3)	305 (72.4)	
above 61	37 (9.6)	39 (9.3)	
***Ethnicity***			0.86 (3), p = 0.461
Sinhala	286 (74.5)	301 (78.2)	
Tamil	27 (7.0)	23 (6.0)	
Muslim	61 (15.9)	56 (14.5)	
Other	10 (2.6)	5 (1.3)	
***Education***			22.55 (2), p = 0.001
No education	27 (7.0)	4 (1.0)	
Primary	95 (24.7)	46 (11.9)	
Secondary	263 (68.3)	335 (87.0)	
***Civil status***			9.00 (2), p = 0.001
Married	345 (89.6)	374 (97.1)	
Unmarried	7 (1.8)	2 (0.5)	
Divorced	33 (8.6)	9 (2.3)	
***Employment***			0.97 (1), p = 0.323
Non-employed	247 (64.2)	260 (67.5)	
Employed	138 (35.8)	125 (32.5)	
***Family indebtedness***			1.08 (2), p = 0.339
No (little or no debt)	214 (55.6)	229 (59.6)	
Yes (significant levels of debt)	171 (44.4)	155 (40.4)	
***Area of residence***			0.15 (1), p = 0.696
Rural	264 (68.6)	269 (69.9)	
Urban	121 (31.4)	116 (30.1)	
Employment type of labor migrant*			
Labourer/domestic maid	249(65.5)	Not applicable	
Services	78(20.5)	Not applicable	
Technical	17(4.5)	Not applicable	
Professional/other	36(9.5)	Not applicable	
***Return frequency of labor migrant***			
Every year	61(15.8)	Not applicable	
Every 2–5 years	110(28.6)	Not applicable	
Never returned/missing	214(55.6)	Not applicable	
***In-bound remittance***			
Every month	144(42.9)	Not applicable	
Every 2–6 months/more	192(57.1)	Not applicable	
***Health-related factors***			
***General health***			9.97 (2), p = 0.001
Excellent	17 (4.4)	19 (5.0)	
Fair	319 (82.9)	349 (91.1)	
Poor	49 (12.7)	15 (3.9)	
***Current illness***			27.94 (1), p = 0.001
Current diagnosed illness	133 (34.7)	70 (18.2)	
No current illness	250 (65.3)	315(81.8)	
***Prevalence of Common Mental Disorders (CMD)***			
Any CMD	74 (19.2)	35 (9.1)	16.56 (1), p = 0.001
Depression	65 (16.9)	30 (7.8)	
Somatoform disorder	22 (5.7)	12 (3.1)	
Anxiety	8 (2.1)	2 (0.5)	

*The categories of migrant worker employment were classified according to the Sri Lanka Bureau of Foreign Employment.

General health was perceived to be three fold poorer in parents of migrant children (12.7%) than compared to the non-migrant families (3.9%), p < 0.001. There were also nearly twice as many migrant parents currently diagnosed with illness (34.7%) than comparative parents from non-migrant households (18.2%) p < 0.001.

Overall prevalence of common mental disorders in adults (depression, somatoform disorder and anxiety) was higher in left-behind parents/caregivers of migrant children [19.2% (95%CI 15.3-23.2)] than the non-migrant parent group [9.1% (95%CI 6.2-11.9)]. Prevalence of depression was doubled in the migrant group (16.9%; 95%CI 13.1-20.6), compared with non-migrant parents (7.8%; 95%CI 5.1-10.5). Prevalence of somatoform disorder (5.7% 95%CI 3.4-8.0) and anxiety disorder (2.1% 95%CI 0.6-3.5) was marginally higher in spouses of migrant families than that of non-migrant counterparts (3.1%; 95%CI 1.3-4.9 and 0.5%; 95%CI 0.02-1.2 respectively).

### Mental health outcome associations with demographic, economic, migration-related and health-related factors

Table 4 describes the unadjusted and adjusted associations between the primary SDQ outcome (any psychiatric diagnosis) with selected child and parental socio-economic and health indicators. In the adjusted analyses, significant associations were observed between child psychopathological outcomes and gender of child, parent/caregiver’s educational attainment and their mental health status.

**Table 4:** **Unadjusted and adjusted associations between SDQ outcome (any psychiatric diagnosis) with child and parent socio-economic and health indicators**

	Unadjusted		Adjusted*	
	Left-behind children	Comparative children	Left-behind children	Comparative children
	OR (95%CI)	OR (95%CI)	OR (95%CI)	OR (95%CI)
**Child age**				
6-11 yrs	1	1	1	1
12-17 yrs	1.06 (0.98-1.13)	0.97 (0.90-1.05)	1.05 (0.98-1.13)	0.97 (0.90-1.04)
**Child gender**				
Male	1	1	1	1
Female	**0.58 (0.36-0.94)**	**0.54 (0.32-0.91)**	**0.60 (0.37-0.98)**	**0.54 (0.32-0.90)**
**Parent gender**				
Male	1	1	1	1
Female	0.70 (0.41-1.17)	0.75 (0.38-1.50)	0.72 (0.43-1.23)	0.80 (0.40-1.60)
**Parent age**				
18-30	1	1	1	1
31-60	1.15 (0.46-2.90)	0.69 (0.32-1.47)	0.83 (0.31-2.20)	0.73 (0.31- 1.72)
61-above	2.37 (0.76-7.34)	1.46 (0.10-25.52)	1.65 (0.50-5.37)	1.61 (0.10-30.36)
**Parent education**				
Secondary education	1	1	1	1
Primary education	1.26 (0.73-2.17)	1.25 (0.61-2.58)	1.19 (0.68-2.07)	1.30 (0.62-2.72)
No education	**3.04 (1.17-7.90)**	2.06 (0.28-14.96)	**2.67 (1.01-7.02)**	2.28 (0.30-17.07)
**Parent employment**				
Employed	1	1	1	1
Unemployed	**1.13 (1.00-1.27)**	1.10 (0.97-1.25)	1.11 (0.98-1.25)	1.09 (0.95-1.24)
**Family indebtedness**				
No (little or no debt)	1	1	1	1
Yes (significant levels of debt)	0.91 (0.56-1.47)	0.62 (0.38-1.02)	0.91 (0.56-1.48)	0.62 (0.37-1.02)
**Parent general health**				
Excellent	1	1	1	1
Fair	0.67 (0.22-1.98)	2.13 (0.44-10.28)	0.57 (0.19-1.74)	2.18 (0.44-10.70)
Poor	1.33 (0.39-4.48)	0.88 (0.10-7.85)	1.04 (0.30-3.61)x	1.05 (0.11-9.65)
**Parent current illness**				
Current diagnosed illness	1	1	1	1
No current illness	0.94 (0.83-1.06)	1.15 (0.97-1.36)	0.96 (0.84-1.08)	1.15 (0.97-1.37)
**Parent mental illness**				
No mental illness	1	1	1	1
Having a mental illness	**1.76 (1.00-3.16)**	**2.56 (1.09-5.97)**	1.63 (0.89-2.96)	**2.44 (1.03-5.80)**

Bold values are significant at p<0.001.

*Adjusted for child age and child gender.

The adjusted odds ratio showed presentation of any child psychiatric diagnosis is 40% less likely in female children as compared to male children in left-behind families [OR 0.60 (95%CI:0.37-0.98), p < 0.05]. A similar association was observed in children from non-migrant families [OR 0.54 (95%CI:0.32-0.90), p < 0.05]. Educational status and employment status of the parents/giver of the child also influenced child psychopathological outcomes. The adjusted odds ratio of having any psychiatric diagnosis was 2.67 times more likely in left-behind children whose parents had not attended school [OR 2.67 (95%CI: 1.01-7.02), p < 0.05].

In children from migrant families whose left-behind parents were unemployed, the likelihood of having any psychiatric diagnosis was 1.13 times higher than those employed [OR 1.13 (95%CI:1.00-1.27)]. However, this result was observed only before adjustment for child age and gender.

## Discussion

This study addresses the question of whether left-behind children of migrant workers are at increased risk for mental health problems and adverse nutritional status. Whilst observational studies cannot determine causality, the evidence suggests overall negative associations at the intersection of labor migration and child mental health, but a weak association with nutritional status.

### Effects on child mental health and wellbeing

A number of researchers have theorized that migration of a parent for extended periods may transform family relationships and functioning [42,43]. For left-behind children, the main concerns centre on how separation from parents affects their social, behavioural and psychological development. In our study, two in every five left-behind children were shown to have clinically relevant child psychiatric disorders. These results suggest that socio-emotional maladjustment and behavioural problems may occur among left-behind children in the absence of a parent. Findings from this nationally representative study corroborates with findings from smaller scale studies conducted in Sri Lanka that showed adverse behavioural outcomes, emotional and conduct disorders in school aged children of female migrant workers [22-24]. The crucial finding was that the psychological impact on families was also observed in families where the father is the overseas migrant worker. Qualitative studies have suggested that it is more challenging to achieve intimacy with children for migrant fathers than mothers [19,44]. Our results also revealed that male left-behind children were more vulnerable to psychopathology. This finding may need further exploration to specifically ascertain gender dimensions of transnational parenting, and measure child resiliency. In the adjusted analyses, significant associations were observed between child psychopathological outcomes and the gender of the child, parental education and their mental health status.

Studies from other Asian countries show mixed patterns of psychological well-being of left-behind children. A study by Graham and Jordan [20] revealed that children of migrant fathers in Indonesia and Thailand are more likely to have poorer scores of psychological well-being than children in non-migrant households. However, this finding was not replicated for children of migrant households in the Philippines or Vietnam. The authors argued for more contextualized understandings in light of results showing divergent mental health outcomes. A study of school children in Philippines by Battistella and Conaco [21] found little evidence that children of migrant families had greater psychological problems than children of non-migrants. A multi-site study in the Philippines (2006) concluded that Filipino children in transnational families were found to be ‘no less anxious or lonely’ than their counterparts in non-migrant families [45]. Acceptance by communities of the ‘normalcy’ of transnational migrant families and for transnational parenting may act as a determinant to reduce vulnerability and enable resiliency of the left-behind child [46]. The anxieties of a child arising from parental separation may be ‘less traumatic’ if the migration experience is shared collectively and is normalized within social structures [46]. It is hypothesized that as international out-migration becomes more normative within high out-migration communities, certain child behavioral problems may decrease [20], with children developing along adaptive trajectories [47]. Since resilience is characterized as consisting of multiple dimensions that may change over time [48], vulnerability may lie upon a continuum that could be exacerbated by extended periods in the ‘left-behind experience’ of a child. Further research, which identifies resilience factors in left-behind children, will therefore be useful.

The Philippines has been recognized for its pro-active policy and program efforts in protecting the rights of its labour migrants and enabling a culture of support to families through a network of civil society and non-governmental organizations [49]. These institutional programs and informal support schemes may also serve to directly and indirectly support the normalization of migration within the social fabric. More research is needed to establish the process and extent to which such programs enable a safe and dignified labour migration experience and how they may be protective for left-behind children.

### Effects on child nutrition

Over a quarter of children aged 6–59 months of migrant households were found to be underweight or severely underweight. The findings of our study revealed that the comparative non-migrant child groups also have a high risk of being underweight (32.7%). These finding are consistent with the underlying nutritional trend in Sri Lankan children that shows an overall prevalence of underweight children in 2009 to be 22.1% [50]. The few studies that describe nutritional outcomes in children left-behind have shown mixed effects; with Gibson (2011) and Nobles (2007) finding an overall negative effect [25,29], while Frank and Hummer [26] attributing a positive effect on child nutrition, especially with high levels of household remittances. The nutritional status of left-behind children may be influenced by a complex inter-play of underlying social determinants and cultural gradients that extend beyond the effects of enhancing purchasing power of food due to remittance income, child care-demands and food-preparation dynamics at household level. Further research is required not only to ‘unpack’ these factors and their associated interrelationships, but also to explore the nutritional impact on child mental health and development [31,32].

### Strengths, limitations and future research directions

A major strength of the study is its representativeness. Previous small scale studies focussed on only male-headed migrant households, derived from a single ethnic group (Singhalese), and from an urban setting within a capital city (Colombo). Our sample was derived from a true cross-section of the left-behind families of migrant workers in Sri Lanka. The generalizability of our findings is therefore enhanced. Our sample was also reflective of the true pattern of work categories published by the SLFBE data: seventy-five percent of labour migrants worked in the ‘unskilled’ labour sector, with professional categories comprising of less than ten-percent of departures.

Our study primarily focused on child psychopathology, however, resilience and protective factors were not adequately explored. Identifying such enabling factors and resilience trajectories in left-behind children is crucial for formulating policies and programs to effectively manage migration and address health and social impact.

Whilst this study provides an insight into how migration effects health status of left-behind children, further research is needed to explore how intra-household power dynamics, transnational parenting, relationship outcomes, and whether male versus female headed households have an effect on child health outcomes. Research is needed to assess how factors such as the duration and frequency of an often cyclical pattern of migration affects health outcomes; how household remittances are actually spent to promote child development outcomes; children’s own experiences/expectations; abuse and violence within migrant families; and, how left-behind families access support services at community level.

Cross-sectional studies may only suggest but not determine causality. Prospective cohort and longitudinal studies are needed to assess if children left-behind truly recover from the experience of parental migration. The re-integration of migrant parents after a long-term absence from child may cause problems due to acculturation issues, family conflicts and re-establishment of livelihoods [51]. The impact of parent–child separation among left-behind children may also need comparison of mental health conditions before and after the separation. From a socio-ecological perspective, those ‘left-behind’ may not only effect migrant children and families but also extend to entire communities [52]. Longitudinal studies are needed to establish if migration actually leads to enhancing health outcomes and aids meaningful social and economic prosperity.

## Conclusions

The findings from this study contribute to an evidence-based approach to developing Sri Lanka’s Migration Health policy and program processors. In our study, two in every five left-behind children were shown to have mental disorders, with a significant burden of socio-emotional maladjustment and behavioural problems. Concerns centre on how separation from parents may effect nutritional, behavioural and psychological development of left-behind children. Community programs to strengthen the capacity of relevant government workers such as public health midwifes (responsible for providing maternal and child health care at domiciliary level), child protection officers, school counsellors and foreign employment agency welfare officers to identify and address social, health and nutrition issues of families left behind are needed. Programs may involve undertaking mapping and vulnerability assessments of migrant families through a coordinated network of such village level workers; development of case management or care plans for left-behind children using community participatory approaches; providing awareness and information to prospective migrant families; and providing guidance for primary caregivers of left-behind children.

As contractual labour migration to places like the Middle East remain a pervasive phenomenon, the impact on children left-behind leaves many unanswered questions. The consequences of long-term migration on family relationship structures, parenting and health vulnerabilities have complex associations that require further longitudinal analysis.

The World Health Assembly resolution on *health of migrants* promotes a ‘safe, dignified and healthy migration’ process for the benefit of both migrants and their families [53]. Though further research is required, this study shows a need to address the social determinants of health affecting migrant families. The finding that almost one-third of the sample were single parent families, that child psychopathology scores were highest in these left-behind families, a growing reliance on elderly care-givers and impacts of trans-national parenting pose complex challenges for policy makers, and raises debate at the nexus of *rights, remittances* and *responsibilities* for both State and ILM. The high levels of malnutrition in many labour sending countries within the developing world and the complex interplay between migrant remittances and child nutrition also form an important yet unexplored policy area. Balancing human rights discourses (for instance, the right of a single mother to migrate for economic reasons), in the context of social and health impact to both families and remittance dependent economies form formidable policy challenges for governments seeking to ‘manage’ migration and development.
